# How Noninvasive Haemoglobin Measurement with Pulse CO-Oximetry Can Change Your Practice: An Expert Review

**DOI:** 10.1155/2013/701529

**Published:** 2013-08-22

**Authors:** Gregor Lindner, Aristomenis K. Exadaktylos

**Affiliations:** Department of Emergency Medicine, Inselspital, University Hospital Bern, Freiburgstrasse, 3010 Bern, Switzerland

## Abstract

Trauma related haemorrhagic anaemia is rarely diagnosed by physical examination alone but typically includes measurement of blood haemoglobin, one of the most frequently ordered laboratory tests. Recently, noninvasive technologies have been developed that allow haemoglobin to be measured immediately without the need for intravenous access or having to take venous, arterial, or capillary blood. Moreover, with these technologies haemoglobin can be continuously measured in patients with active bleeding, to guide the start and stop of blood transfusions and to detect occult bleeding. Recent studies on the accuracy of the devices showed promising results in terms of accuracy of hemoglobin measurement compared to laboratory determination. The present review gives an overview on the technology itself and reviews the current literature on the subject.

## 1. Background

Trauma related haemorrhagic anaemia is rarely diagnosed by physical examination alone but typically includes measurement of blood haemoglobin, one of the most frequently ordered laboratory tests [1, 2]. The need for resuscitation to achieve adequate tissue perfusion is established by the patient's history, on-going bleeding, and clinical signs of hypovolemia. Haemoglobin and haematocrit measurements, the conventional means to confirm hypovolemia, are not always immediately available at the point-of-care and hemodynamic monitoring may not detect relevant blood loss. If treatment is delayed pending laboratory results or diagnostic studies, patient outcome can be affected [3–5]. In particulary in the emergency room, perioperative and critical care settings, rapid and on-going assessment of total haemoglobin is crucial, in order to quantify blood loss and/or the need for transfusion [6]. For example, the rapid determination of blood haemoglobin levels is essential, for the triage of patients in emergency departments [7], and tracking of changes in haemoglobin, to detect occult bleeding, has the potential to be lifesaving during critical care. Therefore, in the hospital setting, there is growing interest in rapid and continuous techniques for measuring haemoglobin and changes in haemoglobin. 

Recently, noninvasive technologies have been developed that allow haemoglobin to be measured immediately without the need for intravenous access or having to take venous, arterial, or capillary blood. Moreover, with these technologies haemoglobin can be continuously measured in patients with active bleeding, to guide the start and stop of blood transfusions and to detect occult bleeding.

## 2. Pulse CO-Oximetry

**Figure 1:**
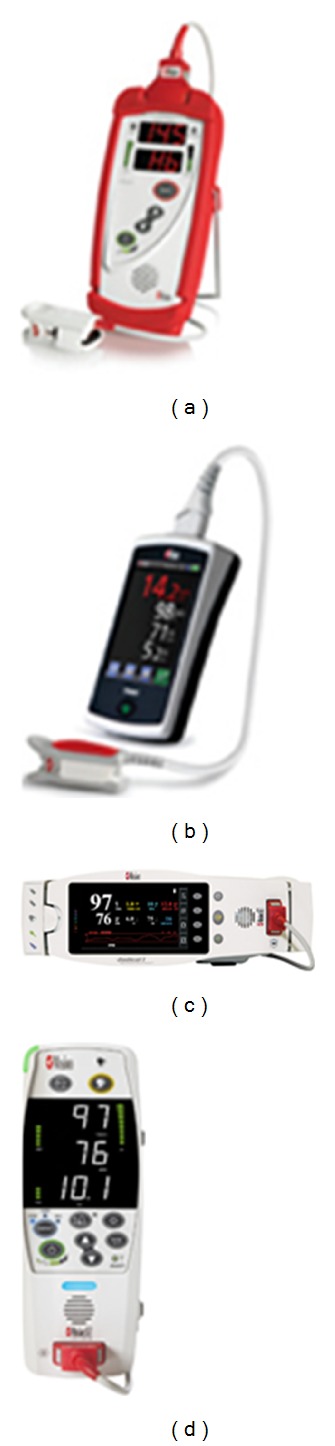
Handheld spot check devices, (a) Pronto; (b) Pronto-7, and continuous monitoring bedside devices, (c) Radical-7 and, (d) Rad-87.

Pulse CO-Oximetry (Masimo Corp, Irvine, CA, USA) is the only commercially available technology that allows for the continuous noninvasive measurement of haemoglobin, referred to as SpHb. This technology uses a multiple wavelength, spectrophotometric sensor that may be an adhesive single use type for continuous monitoring or a reusable finger clip sensor for spot check assessments. Pulse CO-Oximetry allows the noninvasive measurement of carboxyhaemoglobin, methaemoglobin, oxygen content, Pleth Variability Index, along with standard pulse oximetry parameters, oxygen saturation, pulse rate, and perfusion index [9]. SpHb measurement with Pulse CO-Oximetry is available in a number of devices designed for the continuous monitoring at the hospital bedside (Radical-7, Rad-87) or for spot check applications with hand held devices (Rad-57, Pronto) (Figure 1).

Most of the studies published thus far on the performance of SpHb measurement with Pulse CO-Oximetry assess the accuracy of continuous monitoring in surgical patients. Berkow and colleagues [8] investigated the accuracy of SpHb compared to laboratory CO-Oximetry measurement of 130 arterial blood samples from 29 complex spine surgery patients and found an absolute bias and standard deviation of 0.8 ± 0.6 g/dL. Causey et al. [10] studied both surgical and intensive care patients and found a similar bias of 0.29 g/dL. In a study on 44 patients with acute haemorrhage during surgery, Lamhaut et al. compared SpHb and capillary haemoglobin measurement to laboratory determination [11]. The authors obtained a total of 85 measurements, which showed a bias of only −0.02 g/dL (SD 1.39) and a precision of 1.11 g/dL (SD 0.83). However, in comparison to laboratory haemoglobin determination, the percentage of outliers was significantly higher with noninvasive than with capillary measurement. Conversely, when Frasca et al. [12] examined the performance of SpHb in 62 ICU patients providing 471 samples, the bias was 0.0 ± 1.0 g/dL compared to the reference laboratory haematology analyser. However the bias and standard deviation of capillary measurement by HemoCue was 0.3 ± 1.3 g/dL when compared to the reference haematology analyser, significantly higher than SpHb. In general continuous SpHb monitoring accuracy has been found to be comparable to invasive point of care capillary measurement, with some studies showing it to be slightly higher [12] and some studies showing it to be slightly lower [11] when used in the operating room and intensive care unit.

There have also been a few studies published in Emergency Room patients. Sjostrand et al. investigated the accuracy of SpHb using repetitive controls of venous blood samples from 30 patients in a tertiary care emergency room [13]. A total of 242 comparative data pairs were obtained, resulting in a mean deviation of −0.47 g/dL (CI −0.39 to −0.09) for SpHb. After exclusion of 5 patients due to low signal quality, the deviation decreased to −0.24 g/dL (CI −0.39 to −0.09). Chung and colleagues [14] from Inje University Seoul Paik Hospital in Seoul, Korea, studied the accuracy of SpHb compared to laboratory measurements from 217 patients presenting to the emergency department. The correlation coefficient between laboratory haemoglobin and SpHb was 0.81 in all patients indication of good agreement between the two methods of measurement. In a prospective study on 300 emergency patients in France, Gayat et al. compared spot check SpHb to laboratory analysis of venous blood [15]. The absolute mean difference between SpHb and laboratory measurements was 0.56 g/L (confidence interval (CI) 0.41 to 0.69), with a correlation coefficient of 0.80 (CI 0.74 to 0.84). The accuracy of spot check SpHb was also investigated in the outpatient setting by Raikhel [16]. In a prospective observational study, the accuracy of SpHb measurements and capillary measurement of haemoglobin were compared to laboratory haematology analyser measurements from venous blood samples. A total of 156 patients were included in the study, but noninvasive measurement was not possible in 4 patients after two attempts. In the remaining 152 patients, the mean deviation of SpHb from laboratory determinations was −0.5 g/dL (standard deviation (SD) 1.0), with a limit of agreement of −2.5 to 1.5. Results were comparable to haemoglobin determination using capillary blood, with a mean deviation of 0.3 g/dL (SD 1.0) and a limit of agreement of −1.7 to 2.3.

**Figure 2:**
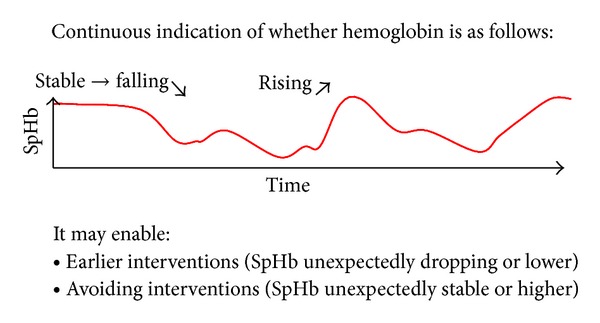
Theoretical model of SpHb monitoring.

**Figure 3:**
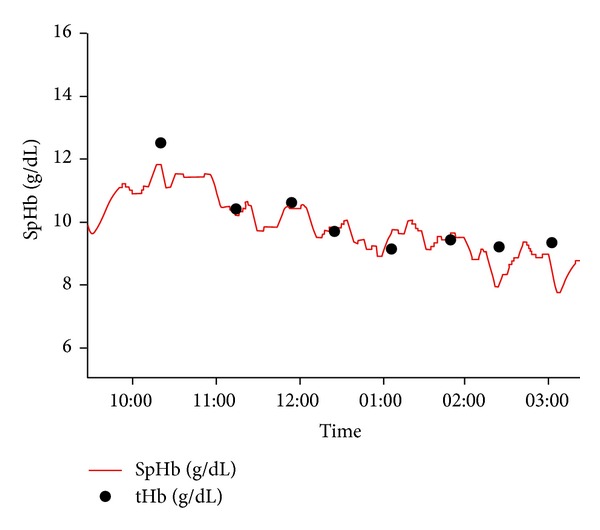
Trend plot of continuous SpHb (red line) and intermittent laboratory haemoglobin values (tHb, black dots) during spine surgery in a 69-year-old female patient [8].

Although the vast majority of published evaluations of SpHb with Pulse CO-Oximetry have been accuracy studies, the true clinical benefit of the technology may be as a trend monitor to detect unexpected changes in haemoglobin, such as with occult bleeding, or to confirm expected changes in haemoglobin as they occur during and after transfusion of red blood cells (Figure 2). Some studies have included an assessment of trend accuracy in the evaluation of SpHb. Berkow examined the magnitude and direction of changes in SpHb when laboratory haemoglobin changed by more than 1.5 g/dL between sequential measurements and concluded that SpHb trended with changes in laboratory haemoglobin but because the average changes were less than 3 g/dL more studies were needed [8].  Figure 3 shows continuous SpHb and intermittent laboratory values during one spinal surgery case in a 69-year-old female. Colquhoun et al. [17] used the four-quadrant plot and the polar plot method to assess trending of SpHb compared to laboratory measurements in 20 patients undergoing major lumbar and low thoracic spine surgery. The four-quadrant plot showed that 94% of SpHb readings outside of the central exclusion zone to eliminate clinically insignificant changes corresponded with the correct directionality. Similarly, the polar plot indicated that 90% of changes in SpHb were within the limits of acceptable trending. Frasca et al. [12] used regression plots of differences in consecutive haemoglobin values reported by SpHb, capillary haemoglobin measurement with HemoCue, and satellite CO-Oximetry measurements compared to central laboratory measurements. SpHb demonstrated the best trending of the three methods with a concordance coefficient of 0.79. Concordance coefficients for satellite CO-Oximetry and HemoCue were 0.74 and 0.76, respectively. 

Indeed, it is the continuous data and trending ability that differentiates this technology from intermittent laboratory or point of care measurements. In fact, the purpose of continuous monitoring is not to replace intermittent laboratory measurements but to improve clinical care by augmenting the data available to the clinician for assessment of the patient. Two preliminary investigations on how SpHb can help guide transfusion decisions, published thus far only as abstracts, support this notion.

The first study, a randomized controlled trial in 327 surgery patients with expected low blood, found that when SpHb monitoring was added to standard care, the frequency of blood transfusions dropped from 4.5% to 0.6% (87% decrease) and the mean units transfused dropped from 0.1 to 0.01 units per patient (90% decrease) (Table 1(a)) [18]. The second prospective cohort study conducted in 106 patients at risk for high blood loss showed that the addition of SpHb monitoring to standard care resulted in a reduction from 1.9 ± 2.3 units to 1.0 ± 1.5 units (47% decrease) in the average number of RBC units transfused and a reduction from 73% to 32% (56% decrease) in the frequency of multiunit RBC transfusions (Table 1(b)) [19]. With SpHb monitoring clinicians were able to initiate transfusions about 9 minutes faster compared to physicians not using the technology, because they did not have to wait for a laboratory haemoglobin value.

## 3. Discussion

The rapid and noninvasive measurement of haemoglobin and the availability of continuous haemoglobin data have the potential to be enormously useful in clinical practice in a variety of situations, such as in trauma, gastrointestinal bleeding, in the perioperative setting, or for guiding blood management during invasive interventions [20, 21]. 

The results from evaluations of SpHb with Pulse CO-Oximetry are promising. In many settings SpHb measurements appear to have similar accuracy as capillary haemoglobin determination when compared to laboratory analysis. Nevertheless, there is room for improvement of the technology (which is on-going), for educating clinician on the best use of the technology and adapting clinical pathways to take advantage of this new tool [16]. In the author's eyes, because the technology is not intended to replace laboratory measurements, it is less important to receive a measurement that exactly mirrors a laboratory value, rather than to provide continuous information regarding the changes or stability of haemoglobin. For the spot check applications, the immediacy of data and noninvasive nature of the device make it ideal for prehospital triage decisions such as choosing the right hospital. At the hospital, this technology has the potential to assist emergency department staff in making triage priorities and in assigning staff and infrastructure. The ease of use of these devices allows for the universal screening of all presenting patients for anaemia which could indicate occult bleeding or other disease processes requiring intervention.

More data from prospective studies is needed to confirm the reliability of this method to guide therapy during surgery or on-going bleeding. Additionally, prospective randomised trials would be desirable to investigate the potential of SpHb monitoring to reduce blood transfusions during surgery or in the intensive care unit.

In conclusion, SpHb by Pulse CO-Oximeter is a promising new medical technology that has the potential to improve the process of care and patient outcomes in many different healthcare settings.

## Figures and Tables

**Table 1:** Differences in red blood cell transfusions when clinicians used standard of care blood management or added SpHb monitoring to guide transfusions in (a) 327 surgery patients with expected low blood loss and (b) 106 surgery patients with expected high blood loss.

(a) Low blood loss surgery (*n* = 327)	Standard care group (*n* = 157)	SpHb Group (*n* = 170)	*P* value
Patients receiving a transfusion *N* (%)	7 (4.5%)	1 (0.6%)	0.03
Total units transfused, *N* (mean)	15 (0.10)	1 (0.1)	0.001

(b) High blood loss surgery (*n* = 106)	Standard care group (*n* = 61)	SpHb Group (*n* = 45)	*P* value

RBC transfusions per subject, mean ± SD units	1.9 ± 2.3	1.0 ± 1.5	<0.001
RBC transfusions per subject receiving a transfusion, mean ± SD units	3.9 ± 1.7	2.3 ± 1.5	<0.01
Transfused patients receiving >3 RBC, % units	73	32	<0.01
Time to transfusion start after need established, mean ± SD min	50.2 ± 7.9	9.2 ± 1.7	<0.001
